# Transcriptional mechanisms of cell fate decisions revealed by single cell expression profiling

**DOI:** 10.1002/bies.201300102

**Published:** 2014-01-28

**Authors:** Victoria Moignard, Berthold Göttgens

**Affiliations:** 1) Department of Haematology, University of CambridgeCambridge, UK; 2) Wellcome Trust – Medical Research Council, Cambridge Stem Cell Institute, University of CambridgeCambridge, UK; 3 Cambridge Institute for Medical Research, University of CambridgeCambridge, UK

**Keywords:** cell fate control, single cell analysis, transcriptional networks

## Abstract

Transcriptional networks regulate cell fate decisions, which occur at the level of individual cells. However, much of what we know about their structure and function comes from studies averaging measurements over large populations of cells, many of which are functionally heterogeneous. Such studies conceal the variability between cells and so prevent us from determining the nature of heterogeneity at the molecular level. In recent years, many protocols and platforms have been developed that allow the high throughput analysis of gene expression in single cells, opening the door to a new era of biology. Here, we discuss the need for single cell gene expression analysis to gain deeper insights into the transcriptional control of cell fate decisions, and consider the insights it has provided so far into transcriptional regulatory networks in development.

## Introduction

Cell and molecular biology have long been studies of averages. Many of our most commonly used techniques report data based on population averages, yet they are widely used to draw conclusions about how individual cells behave. While the study of populations, at least at the molecular level, has until recently been a necessity, it masks a great deal of information about cellular systems, and as a result it influences our interpretation of data. As some of biology's fundamental questions, such as how fate decisions are made by cells, take place at the level of individuals within populations that we know are heterogeneous 1–8, studies of those individuals are long overdue. Furthermore, some populations are so rare – such as those present in the earliest stages of development – that studies of these cells are only possible when techniques are scaled down to a few or single cells.

It has been possible to isolate and culture single cells for some time. Yet, attempts at molecular analysis of mammalian cells have typically been limited in the number of cells and either genes or proteins analysed 7,9–11. Individual mammalian cells are estimated to contain 1–26 pg of RNA, of which mRNA comprises only a few percent of the total 12,13, hence making the analysis of multiple targets challenging. However, recent technological advances have made single cell transcriptomics feasible and affordable, as well as amenable to high-throughput approaches. This enables the analysis of tens to hundreds of genes in hundreds to thousands of cells simultaneously 14–19. New methods vary from microscopy-based, such as single molecule RNA-FISH (sm mRNA-FISH) that can measure a limited number of targets but in potentially large numbers of cells, through RT-qPCR techniques that balance larger numbers of targets with large numbers of cells 12,15,17,20, to microarray and sequencing-based methods. Whereas the latter are able to sample the entire transcriptome, they are currently limited in cell numbers by cost, making RT-qPCR the current method of choice. These methods, their technical aspects and their relative merits have been reviewed in detail previously 12,14–21.

Single cell studies are already revealing a large amount of gene expression heterogeneity between individual cells of specific populations, and as a result, the approaching single cell era may well force us to reconsider much of what we think we know from population studies in light of such molecular heterogeneity. Here, we discuss the need for single cell studies, referring to recent studies of single cells using high-throughput techniques, and their utility for understanding the transcriptional basis of cell fate choices. Transcription factors (TFs) are major drivers of cellular identity and differentiation, as highlighted by their roles in malignancy and as reprogramming factors 22–25. Transcriptional regulatory networks describe the interactions of TFs with cis-regulatory elements on the DNA, and understanding their structure helps us to understand how they function in regulating cellular decision-making 4,26–30. While much has been learned from population studies, detailed analyses of TF interactions in individual cells will revolutionise our understanding of transcriptional regulation in development and also in malignancy.

## Single cell analysis enhances our understanding of transcriptional networks

### Population-average gene expression measurements conceal inter-cellular variation

**Figure 1:**
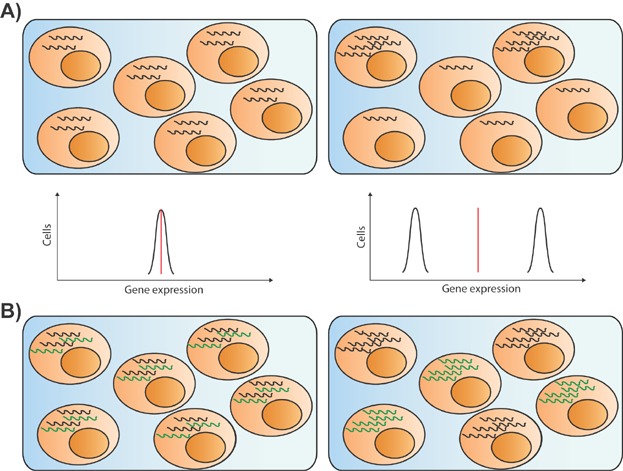
Single cell analysis reveals heterogeneity. A: Single cell analysis can distinguish whether all cells of a population express a similar level of a transcript (top left) or whether a small number of cells account for most of the expression (top right), which cannot be determined from population studies. In single cell studies, a homogeneous population would give a single expression distribution (bottom left) while a heterogeneous population would give a broader distribution, or multiple distributions (bottom right). In population studies, both sets of cells would seem to have the same level of expression (red lines). B: Single cell analysis can reveal whether co-expression observed at the population level actually occurs within the same single cells (left) or not (right).

Transcription occurs as bursts, where a gene is transcribed for a short period followed by a period of inactivity 31,32, and individual genes can exhibit differing burst kinetics. This can result in heterogeneity in expression between individual cells of a population, both in terms of which genes they express and the level of expression. However, the extent to which this variation is stochastic or regulated, and whether it drives or facilitates biological changes, is still open for debate, and is already becoming a major focus of single cell analysis 3,4,33,34. In order to understand which TFs are co-regulated or regulate each other, and how this relates to cell fate, it is important to know whether they are expressed in the same individual cells. Beyond masking functional and molecular heterogeneity, population studies also force us to make assumptions about how individuals behave when extrapolating the data to the single cell level, such that we will assume that all cells of a population express similar levels of a given gene (Fig. 1A). Similarly, when multiple genes are analysed we assume that co-expression at the population level corresponds to co-expression in the individual cells (Fig. 1B). However, studies to date have indicated that often neither of these assumptions hold true at the level of individual cells 12,35,36.

### Gene expression patterns reveal TF regulatory interactions

While lineage commitment may to some extent be a stochastic process, interactions between genes and proteins, as well as the kinetics of expression and degradation, enforce rules on expression that reinforce transcriptional programs once they are activated. Single cell studies offer the potential to identify regulatory links between TFs to generate networks on a larger scale than previously achieved.

**Figure 2:**
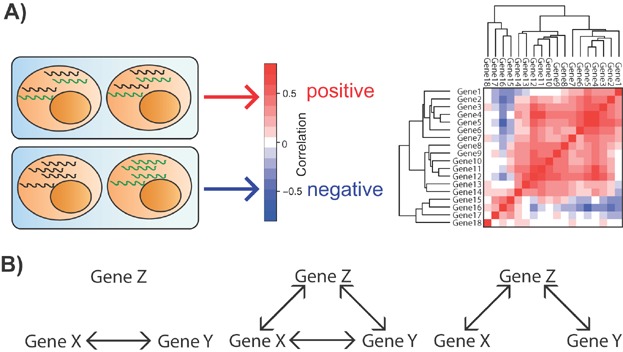
Transcriptional network analysis from single cell gene expression data. A: Single cell expression data can be used to calculate correlations, which describe the likelihood of two genes being expressed at the same time in the same cell. Positive correlations are shown in red and negative correlations in blue. These data can be shown as heatmaps and used to develop hypotheses about transcriptional regulation. B: Partial correlations can be calculated to determine whether the correlation between two factors, X and Y, is direct (left); due to both being regulated by a third factor, Y (right); or a combination of both (middle). These interactions can be validated experimentally using ChIP-seq to identify TF binding to target loci, and reporter assays to show that binding has an effect on gene expression, as well as using perturbation studies to demonstrate that changing the expression of the direct interactor affects expression of the target gene.

Much of the work to date has used correlation analysis across large numbers of individual cells to identify interactions. A positive correlation, where two genes are consistently expressed in the same cells, could indicate that either they share a regulatory mechanism or that one activates the expression of the other (Fig. 2A). Conversely, a negative correlation could indicate that they are independently regulated or antagonistic. Bengtsson et al. 36 analysed the expression of three genes, *Ins1*, *Ins2* and *Actb* at the single cell level after finding that all three were up-regulated in pancreatic beta cells in response to glucose stimulation. While *Ins1* and *Ins2* were up-regulated in the same cells and so had correlated expression, *Actb* was expressed in a separate subset of cells and so was not correlated with the other two genes. This indicates that while *Ins1* and *Ins2* likely share regulatory mechanisms, *Actb* is independently activated in response to the same stimulus. This information is obscured at the population level, resulting in problems in interpretation, and highlighting how putative regulatory interactions determined using population studies may not really occur in individual cells. Furthermore, robust calculation of correlations requires large sample sizes, which single cell RT-qPCR analysis is uniquely able to provide.

Many correlations are generated for even small sets of genes, and not all will represent real regulatory events. ChIP-seq data has been useful in narrowing down the number of correlations that represent true direct regulatory interactions by identifying direct targets of TFs. However, this method is dependent on the existence of data in appropriate cell types, and validation of the function of TF binding events can be time consuming and expensive. The correlations between factors also vary in different cell types due to changes in expression and binding partners. As a result, more efficient computational methods are needed to narrow down the targets for validation and to build networks.

Partial correlations 37 consider whether other genes may interact with the genes of interest and to what extent the correlation between them is the result of interactions with the additional genes rather than a direct interaction (Fig. 2B), as shown in astrocytes for the identification of an interaction network centred around *Vim* 38. Crucially, however, this method can only consider the genes for which measurements were obtained, and networks are generated iteratively from multiple calculations. Other methods are emerging to distinguish direct and indirect interactions more easily and efficiently to generate networks in a single step, and where interactions occur within complex feedback loops. However, these methods still struggle to delineate direct and indirect interactions where the indirect node is not included in the experimental data 39,40. While this is a problem for RT-qPCR data where the number of genes that can be measured simultaneously is limited to hundreds (with TFs numbering into the thousands in mammals) it will become less of an issue as single cell mRNA-seq matures, giving us access to the entire transcriptome 41,42 of individual cells.

Together these studies indicate that in the short period of time in which high-throughput single cell gene expression analysis has been available it has already facilitated significant insights into the biology of single cells that may have implications for disease processes and regenerative medicine. The data so far indicate that cell fate decisions may be at least partly stochastic processes, but that they occur within a defined transcriptional framework governed by transcription factor networks.

## Single cell analysis reveals mechanisms of cellular decision-making

### Cell fate decisions occur in individual cells

#### Single cell analysis reveals key regulators of lineage segregation in early mouse embryos

One of the first single cell studies using microfluidics platforms analysed cells from the mouse zygote through to the 64-cell stage blastocyst 43. Principal components analysis (PCA), a mathematical technique that defines new axes in multidimensional space to capture the variation in data, was able to identify three cell populations based on gene expression, corresponding to the trophectoderm, primitive endoderm and epiblast present in the embryo at the 64-cell stage. From this analysis, two TFs, *Id2* and *Sox2*, were identified as early markers discriminating the different cell types. A negative correlation between *Fgf4* and its receptor was also identified early in the inner cell mass and preceded changes in the transcriptional program 43, providing some insight into the role of signalling in cell fate choices and changes in transcriptional state. When applied to the same data, Gaussian process latent variable model (GPLVM) analysis – an extension of PCA that accounts for nonlinear changes in gene expression – was able to distinguish the primitive endoderm and epiblast at an earlier stage than conventional PCA 44. This indicates how single cell studies are driving the design of better analysis tools.

#### Loss of pluripotency and cell reprogramming involve stochastic and hierarchical phases

In ES cells, heterogeneity in the expression of the pluripotency protein Nanog has been suggested to play a role in the balance between self-renewal and differentiation 5. The effect of loss of *Nanog* on known pluripotency regulatory networks was investigated using a doxycycline-inducible *Nanog* knockdown 45. While removal of *Nanog* resulted in transient up-regulation of differentiation-associated transcripts, there was substantial heterogeneity between cells in the expression of the genes analysed and no subpopulations of similar cells were identified, hence indicating that the earliest stages of differentiation are stochastic 45. This confirms previous assumptions, and is consistent with data from the haematopoietic system 46,47. The pluripotency network is relatively stable if unperturbed by external stimuli, as ground state pluripotency can be tightly maintained in culture with inhibitors of Erk signalling and the beta-catenin/Wnt pathway 48. However, feedback loops between *Nanog* and other pluripotency factors were compromised when *Nanog* was down-regulated, a situation in which different sub-networks activate transiently to push cells toward differentiation. Interestingly, *Nanog* has recently been reported to be biallelically expressed based on both single-cell gene expression analysis and sm mRNA-FISH – heterogeneity in *Nanog* mRNA expression being no greater than for other genes such as *Oct4* 49. This indicates that the heterogeneity previously observed at the protein level may be due to gene-targeting strategies and monoallelic expression or culture conditions. While it is clear that Nanog is down-regulated during the differentiation of the epiblast, what role gene expression heterogeneity in ES cells plays in the down-regulation of *Nanog* and commitment of stem cells is therefore still uncertain.

Reprogramming represents the opposite of this differentiation process, where the ectopic expression of TFs can force cells to revert to a previous state or transdifferentiate to an alternative lineage 22,25,50,51. The production of induced pluripotent stem (iPS) cells by reprogramming, however, is inefficient. A greater understanding of this process at the single cell level could therefore inform the development of better reprogramming strategies. As in the case of differentiation, reprogramming has been viewed as a stochastic process because of its inefficiency 52, but these observations originate from studies of functionally heterogeneous populations in which reprogramming is a rare event. Buganim et al. analysed single cells at multiple time points during reprogramming using either single cell gene expression analysis or sm mRNA-FISH 53 to try to understand the molecular acquisition of pluripotency. PCA identified three groups of cells corresponding to the differentiated cells before reprogramming, iPS cells and an intermediate population that is more heterogeneous both in terms of gene expression and the stage of reprogramming. Early stochastic changes in gene expression were observed that were followed by a more hierarchical stage beginning with the activation of *Sox2*. Bayesian network analysis was then employed to identify linkages between TFs that regulated this hierarchical phase. This was used successfully to predict TFs that could act as reprogramming factors in place of existing protocols.

These single cell studies, in combination with existing literature from population studies, necessarily require changes in how we view cell states such as pluripotency. It has become clear that no single transcriptional state can be associated with, for example the state of pluripotency, even though individual cells may be functionally pluripotent 54. Transcriptional heterogeneity of the pluripotent state may allow the population to respond to a large number of signals, as well as protecting it from perturbations: it has previously been suggested that temporal fluctuations in the levels of key regulators such as *Nanog*, *Stella* and *Rex1* 5–7 influence the ability of ES cells to self renew or differentiate. This has resulted in pluripotency being described in terms of statistical mechanics, a branch of physics that relates the properties of a substance – for example the pressure of a gas – to the stochastic kinetics of its component molecules 54. In the case of cells, the property in question is their transcriptional status. As a population is the average of its components, many configurations or transcriptional states can produce the same functionality.

#### Single cell analysis unravels the haematopoietic hierarchy and identifies commitment events

It is unlikely that these concepts are restricted to ES cells. Nearly 20 years ago, single cell analysis of a limited number of genes in blood cells showed not only that haematopoietic stem cells (HSCs) are heterogeneous in the expression of key regulators – which was subsequently confirmed by us and others 10,46,55,56 – but that they also exhibit promiscuous expression of lineage-affiliated genes 9, which might prime these cells for differentiation. A recent study by Glotzbach et al. 55 aimed to elucidate the relationship between transcriptional and phenotypic variation in HSCs, where subpopulations are known to exist with differing lineage potential. The authors aimed to quantify gene expression heterogeneity and establish whether it constitutes noise around a fixed point or the presence of multiple subpopulations that could correspond to functional states. This is technically challenging, as there is no baseline for gene expression heterogeneity against which to compare expression data. The authors compared CD34^hi^ and CD34^lo^ subsets of HSCs 55, where CD34^hi^ cells have been shown to have a much lower long-term stem cell capacity, and identified nine genes with expression distributions that differed between the two populations and may be important in their differing potentials. The authors used fuzzy c-means clustering 57 to group CD34^lo^ cells on the basis of the expression of these nine genes. This method identifies cells with similar expression and allocates them to clusters to allow their common properties to be identified, but allows each cell to be a member of multiple clusters if it shares properties with several clusters. This identified three clusters of similar sizes. Repeating the analysis on the CD34^hi^ cells that have lower stem cell potential revealed that one cluster was starkly underrepresented compared to CD34^lo^ cells, but this cluster included a similar proportion of HSCs sorted using a different strategy, indicating that this may represent the transcriptional program of true long-term HSCs. Interestingly, expression of some of the nine genes was associated with specific clusters, while others varied between clusters, demonstrating how this analysis can help to discriminate meaningful variation from background noise.

A recent study has also provided insights into how HSCs undergo erythroid commitment. Previous work indicated that the multipotent haematopoietic cell line EML consists of a mixture of cells with differing levels of the surface marker Sca-1, which correspond to differing erythroid and myeloid potentials. Over time, populations sorted for different levels of Sca-1 expression regenerated the original mixed population 8. However, whether individual cells were able to re-establish these mixed cultures had not been shown. Pina et al. 46 used this model system to investigate the transcriptional basis of the erythroid potential of Sca-1^lo^ cells and found that self-renewal and lineage commitment were independent events with correspondingly different transcriptomes. The CD34^−^ compartment of the Sca-1^lo^ cells contained virtually all expression of the erythroid TF Gata1 and had accelerated erythroid differentiation but no culture reconstitution potential, while CD34^+^ cells were multipotent. This insight facilitated transcriptional analysis of cells either side of the commitment boundary. Significant cell-to-cell variation in the expression of a set of erythroid-associated genes was observed around commitment, which resolved to a more homogeneous expression state upon commitment, similar to expression patterns for the same gene set in differentiated erythroid cells 46. While most of the work was performed in cell lines –as it would be difficult to capture cells at the commitment boundary in vivo – similar heterogeneity in the erythroid program was found in primary megakaryocyte-erythroid progenitors compared to more committed erythroid cells. These results indicate that commitment occurs through the independent activation of key regulators in the absence of a coordinated lineage program, with a low probability of transitioning to a committed state due to the requirement for the activation of additional regulators within the same cell. This also suggests that commitment can occur through multiple pathways and that the sequence of events is not entirely fixed, which may have implications for the design of directed differentiation strategies.

Modelling of this data set identified cells close to the commitment boundary and inferred a time course of commitment from static single-cell gene expression measurements 47. Monitoring gene expression changes during commitment again indicated that commitment was mediated by stochastic and independent modulation of key regulators. However, several genes were identified as key in discriminating between self-renewing and committed cells. These included *Gata2*, *Mpl* and to a lesser extent, *Gata1*, with multiple combinations of expression patterns permissive for commitment in modelling experiments and in vivo. In silico perturbation studies indicated that changes in *Gata2* at the mRNA level had the strongest impact on commitment frequency, and permanent activation of *Gata1* increased the likelihood of commitment twofold, which was validated experimentally. However, there was little correlation between the expression patterns of the genes studied, and so the network that regulates commitment is not yet understood.

Single cell analysis is also being employed to delineate pathways of differentiation. While the haematopoietic system is well characterised, there is some disagreement about the ontogeny of the adult system. Analysis of 280 genes, including all commonly used cell surface markers and some important TFs, in multiple cell types of the haematopoietic system 58 showed that in the stem cell compartment levels of the marker CD150 (E-Slam) – which has already been shown to enrich for long-term stem cell capacity compared to CD150^−^ cells 59 – were correlated with the expression of a megakaryocyte-erythroid module of TFs. Furthermore, CD150^+^ cells produced more megakaryocyte-erythroid cells in colony-forming assays. In combination with previous functional studies, this single cell analysis supports the suggestion that megakaryocytic and erythroid cells emerge directly from the HSC, while myelolymphoid cells arise at a later stage 60. This is in contrast to the original model of differentiation in which the HSC gives rise to the CMP, which produces megakaryocytic, erythroid and myeloid cells, and the CLP, which gives rise to the lymphoid lineages 61,62. This study therefore indicates how gene expression analysis can relate transcriptional and cell surface programs, and shows how single cell analysis could be useful in other, less well-defined systems to identify novel surface markers by which to isolate stem cells from contaminating cell types.

### Gene regulatory networks can be characterised using single-cell data

TFs regulate gene expression through interactions with the chromatin at regulatory elements such as promoters and enhancers. Much is known about individual TFs, but while it is clear from functional studies that they act together as part of larger gene regulatory networks 4,27,29,30, less is known about how these networks function to regulate cell fate. Networks are assembled from interlinked motifs, such as positive and negative feedback loops, which perform particular functions. For example auto-regulatory loops can act to reinforce and maintain a factor's expression once activated, while negative auto-regulation results in the repression of a gene by its own product, which can reduce cell-to-cell variation or ‘noise’ in expression 63,64. The connectivity or ‘logic’ of a network determines which factors will be expressed together, so understanding network structure helps us to understand how particular cell states arise, how cells move forward through differentiation and how they decide between alternative fates.

Much of the early work on gene regulatory network construction came from Eric Davidson's studies of the sea urchin 65. Regulatory elements can be defined by examination of conserved regions of the genome, and through analysis of regions bound by TFs in chromatin immunoprecipitation analysis. Perturbation studies, on the other hand, can be used to infer the regulatory relationships or ‘logic’ between factors 28. Our laboratory identified a small network model in haematopoietic cells, in which *Gata2*, *Scl* and *Fli1* are connected to one another through three enhancers 66. Modelling of this triad has shown that it can function as a bistable switch, being either on or off, hence allowing the network to filter noise when responding to external cues 67. A similar triad has been identified between *Oct4*, *Sox2* and *Nanog* in ES cells 68, but, as each factor may have many targets, it can be difficult to identify and validate large networks this way. Networks have also been generated from microarray studies by identifying statistical dependencies between gene products 69,70. However, many systems have inherent heterogeneity, both functionally and in terms of gene expression between individual cells, that is not taken into account using these approaches, and that can only be examined through analysis of individual cells.

#### Regulatory interactions revealed from hundreds of single haematopoietic cells

Several studies have specifically used single cell analysis to characterise gene regulatory networks. We calculated pairwise correlations within a set of 18 TFs in 597 single primary haematopoietic stem and progenitor cells analysed on the Fluidigm BioMark platform 56. This revealed many strong correlations, among which were several known interactions including antagonisms between *Gata1* and *PU.1*, and *Gfi1* and *Gfi1b*. Calculating the significance of correlations and displaying this data as a network allowed us to identify a putative regulatory triad in which *Gata2* is involved in the *Gfi1*-*Gfi1b* antagonism. Importantly, we were able to validate these correlations as direct interactions with an impact on gene expression using ChIP-sequencing and transcriptional assays, suggesting that single cell gene expression analysis is most powerful in combination with existing experimental techniques. Analysis of expression patterns suggested that this network may be important in regulating the exit of cells from the stem cell compartment toward the myelolymphoid lineages. As loss of function of *Gata2* has been implicated in acute myeloid leukaemias 71,72, while inhibition of *Gfi1* could prolong survival in some mouse models of leukaemia 73, the regulatory triad identified from single cell analysis may also be important in the balance between normal differentiation and malignancy. Interestingly, while *Gfi1*−/− HSCs have defects in long-term haematopoiesis due to elevated proliferation and stem cell exhaustion, we identified relatively few HSCs with strong *Gfi1* expression, with the majority expressing the related but antagonistic TF *Gfi1b*. This pattern was also observed in two other recent studies 74,75, indicating that PCR-based single cell studies are highly reproducible, and may require us to revisit and reinterpret existing data. In order to provide robust new insights into developmental and disease processes, this would ideally include non-PCR based assessments of gene expression levels.

Guo et al. 58 also calculated covariances between genes in individual cells from multiple haematopoietic stem and progenitor populations to discover potential regulatory linkages, and integrated their data with existing ChIP-seq studies to exclude interactions where there is no direct TF binding event. An interaction network was generated that highlighted *Gata2* as a core stem cell regulator, and examination of *Gata2* heterozygotes indicated that the stem cell network is sensitive to modulations in the expression of individual TFs: transcriptional changes were identified consistent with the known expression pattern and function of *Gata2* in regulating megakaryocyte-erythroid development. However, this study did not experimentally validate the function of binding events. We found that while TF binding events occur at the *Gfi1b* promoter, this region is not sufficient to drive expression in haematopoietic cells without binding at additional regulatory elements 56. This indicates that not all TF binding events have a functional consequence and so they cannot alone be used to validate regulatory interactions without functional studies.

## Perspectives

The ultimate power of single cell analysis depends to a large extent on the number of cells that can be analysed in parallel. Analysing large numbers of cells simultaneously provides statistical power when calculating correlations and covariances in expression between pairs of genes. It also allows for the capture of rare events that would be hidden in large population studies, such as the commitment of stem cells. This should allow researchers to delineate the steps involved and the molecular mechanisms that underlie them in a way that is not possible when taking average measurements of populations. For example work in multiple systems has indicated that the early stages of lineage commitment are stochastic: some multipotent cells express lineage-affiliated genes that reversibly sway the balance between self-renewal and differentiation 5–7. Furthermore, whether all cells take the same route to a particular committed state, activating a suite of lineage-affiliated TFs, or whether alternative routes exist is also an important question – both in normal development and disease. Studies in this area have the potential to aid the development of directed differentiation strategies for regenerative medicine. While much has yet to be determined, a number of studies, including those discussed above, have begun to make advances towards answering these questions.

Further technological advances are providing more opportunities for studying single cells. Index sorting during FACS allows us to identify the cell surface marker profiles of individual sorted cells. If this information could be correlated with gene expression signatures, it would become possible to isolate particular gene expression states based on surface markers, which may be useful for isolating tissue-specific stem cells 76,77. Protein analysis is also possible at the single cell level, although it has not yet become as popular as gene expression analysis. Mass cytometry uses isotope-labelled antibodies and mass spectrometry to detect over 50 different proteins simultaneously within individual cells 78–80. Analysis pipelines are already in place to visualise relationships between cells and the proteins that drive them 81,82. This methodology has the potential to analyse tens of thousands of cells in a single run, and has been used to study immune signalling in the haematopoietic system 79,80. Microfluidic gene expression platforms are also being utilised for protein analysis, through the use of proximity ligation assays 83. When two different oligonucleotide-tagged antibodies bind to the same protein, the nucleotides are brought into proximity, facilitating a PCR reaction 84 that can be analysed, for example on Fluidigm's BioMark platform.

In the era of ‘big data’, single cell studies are likely to take centre stage, particularly as single cell mRNA and genome sequencing technologies mature. The growing interest in and necessity for studying individual cells is highlighted by the meetings, conferences and courses now dedicated to single cell biology. Many challenges lie ahead, not least for the optimisation of protocols to limit variation in sample collection and processing, but also for the analysis and visualisation of multidimensional data and the production of novel hypotheses. Studies to date have highlighted the insights that can be gained by studying single types of biomolecules, but coupling genomics, transcriptomics and proteomics in individual cells will take systems biology to a whole new level. While this is a rapidly growing field in its own right, the power of single cell studies is in complimenting existing population studies rather than completely supplanting them as it is still necessary to validate findings, and many techniques may remain impractical at the single cell level. However, potentially the biggest challenge for single cell biology will be the inevitable requirement for researchers to forsake established paradigms based on population data in the light of new evidence from the single cell analysis.

## Abbreviations

ChIP-seq: chromatin immunoprecipitation with deep sequencing
FACS: fluorescence activated cell sorting
mRNA-seq: sequencing of messenger RNA
RT-qPCR: reverse transcription quantitative polymerase chain reaction
sm mRNA-FISH: single molecule RNA in situ hybridisation
TF: transcription factor
